# Furloughing*

**DOI:** 10.1111/1475-5890.12242

**Published:** 2020-11-30

**Authors:** Abi Adams‐Prassl, Teodora Boneva, Marta Golin, Christopher Rauh

**Affiliations:** ^1^ University of Oxford; ^2^ University of Zurich; ^3^ Trinity College Cambridge University of Cambridge

**Keywords:** COVID‐19, coronavirus, crisis, recession, short‐time work, furlough, inequality

## Abstract

Over nine million jobs were furloughed in the United Kingdom during the coronavirus pandemic. Using real‐time survey evidence from the UK in April and May 2020, we document which workers were most likely to be furloughed and we analyse variation in the terms on which they furloughed. We find that women were significantly more likely to be furloughed. Inequality in care responsibilities seems to have played a key role: mothers were 10 percentage points more likely than fathers to initiate the decision to be furloughed (as opposed to it being fully or mostly the employer's decision) but we find no such gender gap amongst childless workers. The prohibition of working whilst furloughed was routinely ignored, especially by men who can do a large percentage of their work tasks from home. Women were less likely to have their salary topped up beyond the 80 per cent subsidy paid for by the government. Considering the future, furloughed workers without employer‐provided sick pay have a lower willingness to pay to return to work, as do those in sales and food preparation occupations. Compared with non‐furloughed employees, furloughed workers are more pessimistic about keeping their job in the short to medium run and are more likely to be actively searching for a new job, even when controlling for detailed job characteristics. These results have important implications for the design of short‐time work schemes and the strategy for effectively reopening the economy.

## I Introduction

The coronavirus outbreak has brought about a severe economic recession. With lockdown measures and business closures in place to contain the spread of the virus, many businesses have seen their activities coming to a halt. This has led to a sharp rise in unemployment rates in many countries affected by the coronavirus pandemic. To counteract the economic consequences of the current crisis and to partially shield workers from the economic downturn, many countries have introduced or expanded existing furloughing or short‐time work (STW) schemes.^1^ In the UK, the government launched the Coronavirus Job Retention Scheme (CJRS) on 20 April 2020. The scheme allows employers to furlough workers for a minimum of three weeks, with the government contributing 80 per cent of employees' salaries. By 14 June 2020, more than nine million jobs had been furloughed under the CJRS, for a total value of claims of more than £20 billion.^2^

While a third of UK employees have been enrolled in the CJRS, little is known about the operation and effectiveness of the policy. It is thus difficult to assess when the scheme should optimally end, and the degree to which furloughing should feature in the policy response to any future waves of infection. Further, the CJRS leaves a lot of room for employer discretion in the terms on which workers are furloughed. Whether the exercise of such discretion is reducing or exacerbating existing dimensions of labour market inequality is important for the design of policies to support the economic recovery.

In this paper, we use survey data that we collected on two independent samples of workers to shed light on the operation of the UK furloughing scheme. We find large variation in the share of workers that have been furloughed across, but also within, occupations and industries. Women have been significantly more likely to be furloughed than men doing the same type of job. There is evidence that childcare responsibilities play an important role in explaining this gender gap. Mothers are 10 percentage points more likely than fathers to have initiated the decision to be furloughed, as opposed to the decision being ‘fully’ or ‘mostly’ the employer's decision, when controlling for a rich set of job characteristics. However, we find no gender gap in the furlough decision amongst childless workers.

We find that ‘not all workers are furloughed equally’, and we document differences in the terms under which workers are put on furlough, including whether employers have agreed to top‐up their employees' salaries beyond the state contribution. Women and those on low incomes are less likely to have had their wages topped up beyond the 80 per cent provided by the government. We find that the majority of workers have continued to do some work while furloughed without being formally rotated back into employment. Amongst furloughed workers who can do at least 50 per cent of their job from home, only 17 per cent report working zero hours and their work hours are only 25 per cent lower than they were in February.^3^

Finally, we examine workers' expectations about future unemployment. We find that workers' perceived probability of losing their job before August is 28 per cent, but that furloughed workers perceive a 15 percentage point higher likelihood of job loss in the coming months. We also show that more pessimistic expectations increase on‐the‐job search, and that having been furloughed further increases the probability of job search by 3 percentage points.

Our results have important implications for the design of the UK furloughing scheme, and STW policies more broadly. First, STW schemes should allow employees to work on a part‐time basis. Indeed, it is odd that the UK scheme originally ruled out this possibility, given that such flexibility is a key reason to prefer STW schemes over recall unemployment. It is very rare for workers to report that they can do precisely zero of their work tasks from home,^4^ and the majority of workers have continued to do some work while on furlough. Perversely, firms breaking the terms of the scheme in this way have likely been welfare‐improving, although it has introduced horizontal inequity between compliers and non‐compliers; firms will have had more flexibility in maintaining essential business activities and the rate of human capital depreciation should have slowed.

The duration of support is a crucial parameter of STW schemes. These policies should be active long enough to prevent inefficient layoffs from firms in temporary hardship. However, they should not subsidise low‐productivity matches indefinitely and thereby hinder efficient labour market reallocation.^5^ Our results suggest that there is another dimension to consider. Crucially, the duration of the furloughing scheme should be sensitive to continued disruption in schooling and childcare. Mothers have been more likely to request to be furloughed. There is a real risk that these women could be forced out of the labour market if the furloughing scheme ends without viable childcare options being available. Our results also suggest the need for flexibility in the removal of the scheme across different occupations. Furloughed workers who can do a large proportion of their jobs from home are relatively pessimistic about their chance of keeping their job. For these workers, social‐distancing measures are unlikely to be the only reason for a low‐productivity match and they should not be prevented from moving to more viable firms.

Finally, a return to work outside the home provides more opportunities for catching and transmitting the virus. We find wide variation in the willingness to return to work from furlough. Workers without access to employer‐provided sick pay have significantly lower willingness to pay to return to work from furlough. Worryingly, we find that workers without sick pay are significantly more likely to continue to work with mild coronavirus symptoms. The UK has one of the least generous statutory sick pay schemes in Europe, which was described as ‘manifestly inadequate’ by the European Committee of Social Rights (2017). Our results suggest that the provision of more generous sick pay could help to support the economic recovery by encouraging workers to return to work while infection rates remain above zero, and supporting sick workers to take time off work when they pose a risk to others.

Our paper contributes to several strands of the literature. First, it contributes to the literature on the importance of STW schemes to buffer economic shocks^6^ and to the growing body of literature documenting the immediate economic impact of the COVID‐19 pandemic in the UK.^7^ Other research using data collected before the crisis has discussed channels through which the current crisis may affect workers differently depending on their gender and occupation.^8^ Our results are consistent with Andrew et al. (2020), who also find big differences in the labour supply of mothers and fathers over the pandemic. Finally, our paper contributes to the literature on the positive externalities arising from sick pay coverage.^9^ We show that even amid the pandemic, when the importance of social distancing and self‐isolation was particularly salient, workers without sick pay were significantly more likely to work when sick and that workers without sick pay are less willing to return to work from furlough.

## II Institutional features and design choices

### 1. Policy motivation

STW schemes subsidise labour hoarding by firms. They allow firms to reduce employees' hours rather than firing them, with the government stepping in to smooth workers' salaries. STW schemes have been a key pillar of countercyclical policy in several countries for many years. Germany, for example, has one of the oldest and most comprehensive STW programmes in the world.^10^ The German *Kurzarbeit* scheme allows firms to reduce their employees' hours for up to 12 months. The government replaces 60 per cent of forgone net monthly earnings (up to a cap) for single workers in order to shield them from the financial impact of the fall in hours.^11^ Similar schemes exist in many other European countries and in some US states.^12^

Why implement a STW policy rather than insuring workers directly through unemployment insurance schemes? STW schemes aim to preserve worker–firm matches in the face of temporary negative shocks; firm‐specific human capital and hiring costs mean that it can be efficient to keep a worker–firm match intact in periods of low productivity. However, liquidity constraints and/or commitment issues limit the degree to which firms can do this in practice.^13^ This provides a role for governments to subsidise labour hoarding and reduce inefficient layoffs. While firms can fire workers and rehire them when business conditions improve, commitments to recall workers are generally not credible. In their model, Gregory, Menzio and Wiczer (2020) emphasise the importance of furloughing to avoid job ties being cut for workers who could take years to find stable jobs. STW schemes also allow much more flexibility than so‐called temporary or recall unemployment; most STW schemes allow employees to work on a part‐time basis, helping to maintain essential business activities and preventing depreciation of human capital.

In an aggregate crisis, STW schemes can relieve the public administration of some of the burden of allocating funds quickly to those in need. In the US, for instance, the reports of long delays in payments and long queues in front of public offices during the COVID‐19 pandemic are plentiful.^14^ As STW schemes can operate directly through employers, applications can be coordinated around a smaller number of agents and the paperwork burden on workers can be minimised.

To evaluate the overall effects of STW schemes, there are several factors to consider. First, does the scheme reduce inefficient separations? Evidence from the Great Recession suggests that some STW policies can have large positive effects on employment: Giupponi and Landais (2020) and Cahuc, Kramarz and Nevoux (2018) exploit variation in eligibility rules to show that the Italian and French STW schemes, respectively, have strong positive employment effects on liquidity‐constrained firms. However, the devil is in the details; schemes must likely provide timely payments and extend for the duration of the shock if liquidity‐constrained firms are to retain workers into a downturn. It is also important to consider whether all types of labour are covered by the scheme, in order to prevent inefficient substitution between different workers.

Second, how large are moral hazard effects? Moral hazard can take many forms in this context. Firms might take advantage of the scheme by requiring workers to put in their usual hours with their wages subsidised by the state. In the present crisis, this is more likely to be a pressing issue in occupations where working from home is easier. Evidence of significant downturns in production as a condition for wage subsidies could help limit such behaviour and is used in practice in some countries (e.g. Germany). Alternatively, firms may accept subsidies and still lay off workers. Take‐up should, therefore, be made conditional on retaining workers; the precise terms in which this obligation is made varies across countries.^15^

Third, do STW schemes prevent workers from moving to higher‐productivity firms? By subsidising lower‐productivity matches, STW schemes could prevent workers from leaving failing firms quickly and thus hinder efficient labour market reallocation. Giupponi and Landais (2020) show that this effect is especially important for persistent shocks. In the present context, this question cannot be evaluated at this stage, given that the pandemic remains active and the persistence of the downturn remains unknown.

Finally, many schemes leave room for firm discretion regarding how to allocate reductions of hours across their workforce, whether wages should be topped up beyond government subsidies, and the removal of non‐wage work benefits. As far as we are aware, there is no existing evidence on heterogeneity in the terms on which workers are enrolled in STW schemes. The consequence of employer discretion on these margins for labour market outcomes is an empirical question that we hope to shed light on in this paper.

### 2. The UK Coronavirus Job Retention Scheme

In the UK, on 20 March 2020 the government announced a new STW scheme to protect jobs – the Coronavirus Job Retention Scheme (CJRS). The operation and expected duration of the scheme have been continuously revised over the crisis. It closed to new applications on 30 June 2020.^16^ There are two particularly noteworthy features compared with other European STW schemes: tight restrictions on flexible working and uncertainty over the duration and generosity of the scheme.

The UK scheme initially placed severe restrictions on work for enrolled employees. Until 1 July 2020, workers on the scheme had to be furloughed and do *no* work for their employer for at least three weeks in each four‐week period.^17^ In return, the government paid 80 per cent of employees' wages, up to a maximum of £2,500 per month. This stands in contrast to the STW schemes in Italy, France and Germany, which allowed for flexible reductions in hours. In principle, flexible reductions in hours seem preferable as a minimum number of hours may be necessary to sustain critical business operations and to prevent depreciation of individual and firm‐specific human capital.

On 12 June, the UK scheme was revised to permit ‘flexible furloughing’ from the beginning of July. From 1 July, employers have been able to bring furloughed employees back to work and claim subsidies for typical hours not worked by an employee (with employers paying for hours that are worked). However, note that this arrangement is only available for workers who were previously ‘fully’ furloughed. The introduction of short‐time work within the scheme was previously announced to be available from 1 August but was brought forward by a month to facilitate a return to work with the easing of lockdown measures. From 1 August, employers are also required to make gradually increasing contributions towards labour costs.^18^

As this discussion highlights, firms have faced considerable uncertainty about the length, generosity and design of the UK scheme. When announced, the UK scheme was guaranteed to last for four months, until the end of June 2020. At the time of writing, the scheme has been extended until the end of October. It remains unclear whether the scheme will operate beyond this point, and if so, under what terms. It is also worth noting the initial delay in payments. While the scheme was announced in late March, the portal to facilitate payments to firms did not open until the end of April.

## III Data

To study variation in the characteristics of workers furloughed, and heterogeneity in the terms under which they have been furloughed, we collected real‐time survey data on large geographically representative samples of UK workers.^19^ The data were collected by a professional survey company; all participants were part of the company's online panel and participated in the survey online.^20^ We collected two waves of survey data that included detailed information on furloughing. The first wave of data (N= 4,931) was collected on 9–11 April 2020 (approximately two weeks after the introduction of lockdown measures in the UK). The second wave (N= 4,009) was collected on 20–21 May 2020.^21^ To be eligible to participate in the study, participants had to be resident in the UK, be at least 18 years old, and report having engaged in any paid work during the previous 12 months. While our surveys targeted individuals who were or had been engaged in any type of paid work, including self‐employment, in the analysis we restrict the sample to respondents who reported being in paid work in February 2020, and who were (had been) employees in their main (last) job.

The samples were selected to be representative in terms of region. Table A.1 in the online Appendix shows the distribution of respondents across regions in the UK and the comparison with the national distribution of individuals across the different regions, separately for each survey wave. As can be seen from this table, the distributions are very similar. We compare the characteristics of the respondents in our sample to a nationally representative sample of the economically active population in the UK. Table A.2 shows the demographic characteristics of our samples and of economically active workers in the Labour Force Survey (LFS) in the second quarter of 2019.

### 1. Economic activity and furloughing

In our surveys, we asked respondents about the number of jobs they had in February 2020 and in the week before the survey date. Respondents were asked to think about jobs they had other than completing surveys and were told to count jobs from which they were furloughed as a job. Respondents who worked at least one job in February were then asked for their typical weekly hours in February. Respondents who had at least one job in the survey reference week were asked how many hours they worked last week.

Workers who had at least one job in the week before data collection were asked detailed questions about their main job, including whether they were furloughed.^22^ Note that we asked *all* employees if they had been furloughed; that is, we did not condition this question on whether a respondent reported zero work hours last week to allow us to analyse compliance with the terms of the CJRS. This is in contrast to some other UK labour market surveys, which have conditioned their question about furloughing on a report of zero work hours in the survey reference week.^23^

### 2. Furloughing terms

We collected information on the terms under which workers have been furloughed. In the April survey, we asked respondents whether their employer had topped up their wage beyond the 80 per cent paid by the government. We also collected information on whether employers were still asking respondents to work, distinguishing between those who were being formally rotated back into work and those who were being asked to work in violation of the terms of the scheme.^24^

In our May survey, we asked questions about whether the worker or their employer made the decision to go on furlough and whether a respondent wanted to return to work. Specifically, we asked about the degree to which furlough was the employer's or respondent's decision on a five‐point scale ranging from ‘Fully [the employer's] decision’ to ‘Fully [the respondent's] decision’. Respondents who were currently furloughed in the May survey were also asked whether they would prefer to go back to their usual work hours for 80 per cent of their usual salary.

### 3. Economic impacts

Furloughing is only effective if it limits inefficient separations. To obtain a better sense of how individuals perceived their future labour market outcomes, we asked respondents how likely they thought it was that they would lose their job before 1 August 2020, on a scale of 0–100 per cent chance. In our second survey, we also asked respondents how likely it was that they would look for a new job in the next 12 months, again on a scale of 0–100 per cent chance.

## IV Who was furloughed?

**FIGURE 1:**
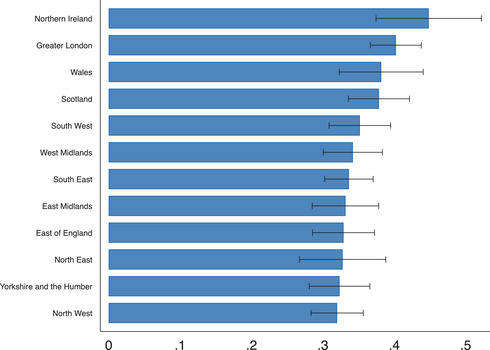
Share of furloughed workers by region *Note*: The horizontal bars show the average share of employees who were furloughed on the survey date for each region. The black bars represent 95 per cent confidence intervals. Survey responses for the April and May survey waves are pooled in this figure.

In our sample of UK employees, 35 per cent of those in work in February report being currently furloughed from their main job. This figure is consistent with the best available UK administrative records. Official records show that 9.4 million claims were made to the furloughing scheme by late June 2020. Assuming that each worker is only furloughed from a single job, this corresponds to 34 per cent of employees.^25^ In Figure 1, we exhibit the share of furloughed workers by region. The share of workers furloughed across regions varies from 32 per cent in the North West to 45 per cent in Northern Ireland.

**FIGURE 2:**
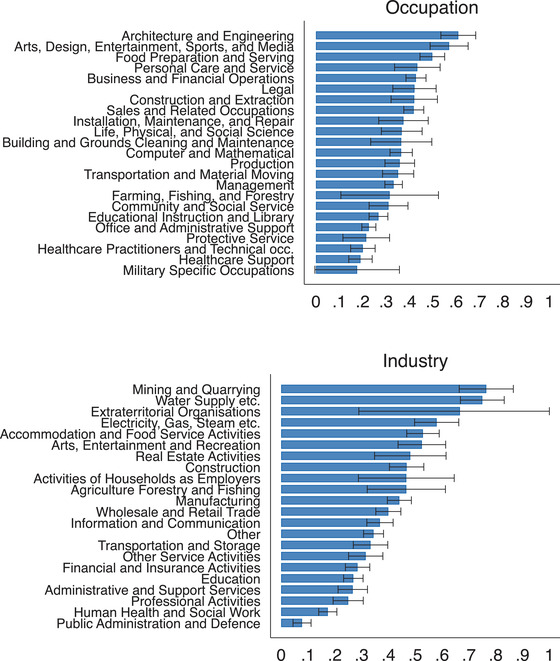
Share of furloughed workers by occupation and industry *Note*: The horizontal bars show the average share of employees who were furloughed on the survey date for each occupation (top) and industry (bottom). The black bars represent 95 per cent confidence intervals. Survey responses for the April and May survey waves are pooled in this figure.

There is a lot of variation in the extent to which employers made use of the furloughing scheme across both industries and occupations. In Figure 2, we report the share of furloughed employees by occupation (top) and industry (bottom) when pooling our April and May survey waves.^26^ For occupations, the share of employees who reported having been furloughed ranges from 61 per cent for ‘Architecture and Engineering’ to 19 per cent for ‘Healthcare Support’. Across industries, 76 per cent of those employees in February working in ‘Mining and Quarrying’ report having been furloughed, against a figure of 8 per cent for ‘Public Administration and Defence’.

**FIGURE 3:**
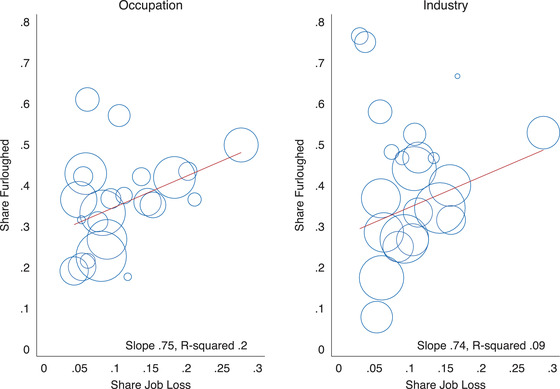
Share of workers furloughed and share that have lost their job across occupations and industries *Note*: Each circle represents either an occupation or industry, with the size proportional to the number of survey respondents who report that either their current or last job was in that occupation or industry. The line gives the line of best fit. Survey responses for the April and May survey waves are pooled in this figure.

One might have expected the share of furloughed employees to have been greatest in ‘Accommodation and Food Service Activities’ given that this industry has been particularly affected by sector‐specific lockdowns. While 53 per cent of employees working in this industry report being furloughed, which is higher than average, job loss has also been particularly high (29 per cent). In contrast, in many utility industries (e.g. ‘Water Supply etc.’, ‘Electricity, Gas, Steam etc.'), a large proportion of workers have been furloughed but few have lost their job.^27^ Figure 3 shows the relationship between the share of employees that lost their jobs and the share that were furloughed across occupations (left) and industries (right). While there is a significant positive relationship between the rates of job loss and furlough, there is considerable heterogeneity in the furloughing rate amongst occupations and industries with similar levels of job loss.

**FIGURE 4:**
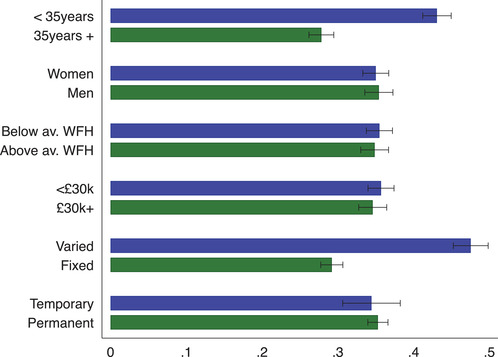
Share of furloughed workers by individual and job characteristics *Note*: The graph shows the share of workers that are currently furloughed by different individual and job characteristics. Black bars represent 95 per cent confidence intervals. Survey responses for the April and May survey waves are pooled in this figure. ‘Below av. WFH’ are employees who can do less than average tasks from home, while ‘Above av. WFH’ are employees who can do more than average tasks from home. ‘<£30k’ refers to respondents with a yearly gross individual income below £30,000 in 2019, while ‘£30k+’ refers to those earning more. ‘Varied’ refers to respondents with variable‐hour contracts, while ‘fixed’ refers to those with fixed‐hour contracts.

Turning to differences in the probability of being furloughed by background and job characteristics, Figure 4 shows that workers with unstable work arrangements were significantly more likely to be put on furlough. In particular, 48 per cent of workers with variable hours were put on furlough by May 2020, against a corresponding figure of 29 per cent of workers with fixed‐hour contracts. Workers under the age of 35 were significantly more likely to be put on furlough by May 2020 compared with workers aged 35 or above.

In columns (1)–(3) of Table 1, we consider which workers were furloughed within the framework of a linear probability model (LPM). In column (1), we see that occupation and industry are important determinants of whether an employee is furloughed or not: together with region and time fixed effects, they explain 10 per cent of the variation in furloughing. Job characteristics are important predictors of furloughing.^28^ Throughout all specifications, workers on variable‐hour contracts and those who are paid by the hour are much more likely to have been furloughed, while those who can do a greater percentage of their work tasks from home have been less likely to be furloughed. Controlling for job characteristics, as well as a broad set of fixed effects, we find that women were 3 percentage points (pp) more likely to have been furloughed compared with men. Moreover, workers on variable‐hour contracts, if either the firm or the worker decides on the schedule, are also significantly more likely to have been furloughed. The probability of being furloughed is u‐shaped in terms of age, with young workers below the age of 30 being the most likely to have been furloughed.

**TABLE 1:** Furloughing probability: job and individual characteristics

	*LPM*			*Multinomial logit*	
	Furloughed			Furloughed	Lost Job
	*(1)*	*(2)*	*(3)*	*(4)*	*(5)*
Age:					
30–39		−0.1312***	−0.0806***	−0.4575***	−0.3773***
		(0.0172)	(0.0171)	(0.0845)	(0.1324)
40–49		−0.1984***	−0.1164***	−0.6491***	−0.4955***
		(0.0183)	(0.0187)	(0.0961)	(0.1464)
50–59		−0.2695***	−0.1642***	−0.9872***	−0.6940***
		(0.0200)	(0.0206)	(0.1187)	(0.1703)
60+		−0.1982***	−0.1097***	−0.6712***	−0.7919***
		(0.0305)	(0.0306)	(0.1620)	(0.2564)
University degree		−0.0382***	−0.0038	−0.0107	−0.0100
		(0.0128)	(0.0138)	(0.0738)	(0.1129)
Female		−0.0239*	0.0279**	0.2027***	0.3127***
		(0.0128)	(0.0136)	(0.0721)	(0.1132)
Income 2019 (£10,000s)			0.0063**	0.0298**	−0.0034
			(0.0029)	(0.0145)	(0.0263)
Temporary contract			−0.1262***	−0.3080***	0.9074***
			(0.0223)	(0.1154)	(0.1389)
Variable hours (worker)			0.0758***	0.4029***	0.2638*
			(0.0177)	(0.0890)	(0.1415)
Variable hours (firm)			0.0682***	0.3822***	0.1488
			(0.0209)	(0.1051)	(0.1505)
Non‐salaried contract			0.1181***	0.5582***	0.1051
			(0.0161)	(0.0793)	(0.1211)
Work from home			−0.0554***	−0.6065***	−1.8480***
			(0.0201)	(0.1116)	(0.1851)
No paid sick leave			−0.0439***	0.0295	0.8219***
			(0.0167)	(0.0879)	(0.1136)
Constant	0.4984***	0.5848***	0.5317***	0.3591	−1.6383**
	(0.0854)	(0.0275)	(0.0906)	(0.3965)	(0.6781)
Observations	5,522	5,540	5,476	5,476	
$R^{2}$	0.1008	0.0465	0.1350		
Region F.E.	Yes	Yes	Yes	Yes	
Wave F.E.	Yes	Yes	Yes	Yes	
Occupation F.E.	Yes	No	Yes	Yes	
Industry F.E.	Yes	No	Yes	Yes	

*Note*: Standard errors in parentheses. ^***^ p<0.01; ^**^ p<0.05; ^*^ p<0.1. Columns (1)–(3) report linear probability models where the dependent variable is a dummy variable that takes the value of 1 if the respondent reports that they are currently furloughed from their main job, and 0 otherwise. Columns (4) and (5) report the coefficients of a multinomial logit model where the omitted category is ‘employed and not furloughed’.

These models ignore the fact that workers can be in three states: furloughed, employed and not furloughed, and not in work. Columns (4) and (5) analyse worker outcomes in a multinomial framework, where ‘employed and not furloughed’ is the omitted category. Similar patterns arise. Notably, women are significantly more likely to have been furloughed or lost their job. Younger workers and those employed on variable‐hour contracts are less likely to be in non‐furloughed employment. While workers on temporary contracts have been less likely to be furloughed, they are more likely to have been laid off. Those on higher incomes are more likely to have been furloughed relative to remaining in employment or losing their job.

## V Furloughing terms

Heterogeneity in the terms on which workers are furloughed arises along several dimensions. Did the worker or employer initiate the decision to be furloughed? Are worker incomes ‘topped‐up’ by employers beyond the 80 per cent paid for by the government? Do employees continue to work while furloughed even though it is against the terms of the scheme?^29^

**FIGURE 5:**
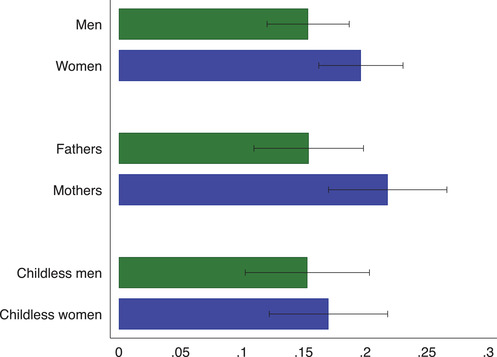
Share of furloughed employees who asked to be furloughed *Note*: The graph shows the share of currently furloughed employees who initiated furloughing. We construct a binary variable that takes the value of 1 if the respondent reports that they had an equal say in the furloughing decision, or the furloughing was initiated mostly or fully by them. Mothers or fathers are defined as respondents who have at least one child living in the household. The sample is restricted to respondents to the May survey wave.

Consider first the decision to be put on furlough. We asked respondents to identify whether the decision to be furloughed was ‘fully [their] employer's decision’ to ‘fully [their] decision’ on a five‐point scale.^30^ Figure 5 shows whether an employee had at least an ‘equal say’ in the decision to go on furlough by gender and by whether the respondent has children. We construct a binary variable that takes the value of 1 if the respondent reports that they had an equal say in the furloughing decision, or the furloughing was initiated mostly or fully by them. Women are more likely to have initiated furloughing and this is mainly driven by women with children at home who are much more likely to have initiated furloughing than men with children. These results highlight an important gender gap in the impact of the pandemic and are consistent with findings that mothers are spending significantly more time on childcare activities than men during the pandemic, at the expense of paid work time.^31^

In Table 2, we look at which workers are more likely to have initiated the furloughing in a regression framework. In column (1), we find that women are 4 pp more likely to have asked to be put on furlough, compared with men. The coefficient on the female dummy remains stable when controlling for occupation and industry fixed effects, as well as a number of job characteristics (column (2)). We then examine whether childcare responsibilities might affect a worker's decision to initiate furloughing. When restricting the sample to parents (columns (3) and (4)), we find that women are almost 10 pp more likely to initiate the furloughing, whereas we do not find a gender gap in who initiates furloughing in the group of respondents without children (columns (5) and (6)).

**TABLE 2:** Who initiated furloughing?

	All		Parents		No children	
	*(1)*	*(2)*	*(3)*	*(4)*	*(5)*	*(6)*
Age:						
30–39	−0.0467	−0.0291	−0.0797**	−0.0501	0.0151	0.0209
	(0.0316)	(0.0315)	(0.0403)	(0.0431)	(0.0571)	(0.0539)
40–49	−0.0277	0.0246	−0.0333	0.0115	−0.0217	0.0401
	(0.0371)	(0.0372)	(0.0502)	(0.0510)	(0.0533)	(0.0550)
50–59	−0.0606	0.0155	−0.1148*	−0.0417	−0.0140	0.0251
	(0.0409)	(0.0422)	(0.0647)	(0.0751)	(0.0577)	(0.0610)
60+	0.0253	0.1064*	0.3032	0.4504**	0.0290	0.0680
	(0.0572)	(0.0594)	(0.1988)	(0.1988)	(0.0653)	(0.0692)
University degree	0.0293	0.0338	0.0549	0.0387	0.0047	0.0192
	(0.0257)	(0.0272)	(0.0338)	(0.0392)	(0.0405)	(0.0425)
Female	0.0432*	0.0537*	0.0711**	0.1048***	0.0240	−0.0176
	(0.0254)	(0.0278)	(0.0351)	(0.0377)	(0.0382)	(0.0445)
Income 2019 (£10,000s)		0.0068		0.0066		0.0142
		(0.0058)		(0.0077)		(0.0110)
Temporary contract		0.0273		0.0662		0.0224
		(0.0445)		(0.0614)		(0.0676)
Variable hours (worker)		0.0817**		0.0545		0.1924***
		(0.0342)		(0.0425)		(0.0666)
Variable hours (firm)		0.1394***		0.1437***		0.1277**
		(0.0368)		(0.0512)		(0.0566)
Non‐salaried contract		0.0509*		0.0132		0.0719
		(0.0283)		(0.0398)		(0.0456)
Work from home		−0.0174		0.0029		−0.0676
		(0.0403)		(0.0632)		(0.0566)
No paid sick leave		−0.0624**		0.0016		−0.1213***
		(0.0313)		(0.0549)		(0.0403)
Constant	0.0984**	0.2809	0.1051	0.2381	0.0894	0.9117***
	(0.0501)	(0.2019)	(0.0691)	(0.2055)	(0.0746)	(0.1140)
Observations	968	963	537	533	431	430
$R^{2}$	0.0203	0.1248	0.0560	0.1636	0.0244	0.2122
Region F.E.	Yes	Yes	Yes	Yes	Yes	Yes
Occupation F.E.	No	Yes	No	Yes	No	Yes
Industry F.E.	No	Yes	No	Yes	No	Yes

*Note*: OLS regressions. Standard errors in parentheses. ^***^ p<0.01; ^**^ p<0.05; ^*^ p<0.1. The sample is restricted to furloughed respondents to the May survey wave. The dependent variable is a dummy variable that takes the value of 1 if the respondent had an equal say in the decision to initiate the furloughing or if the furloughing was mostly the respondent's decision. The dependent variable takes the value of 0 if the furloughing was initiated fully or mostly by the employer.

We also find that those on variable‐hour contracts are more likely to have initiated the decision to be furloughed. This is especially so for those where the employer, rather than the worker, has the discretion to determine working hours: those with employer‐determined hours are 14 pp more likely to have initiated furlough than those with a fixed‐hours schedule. This does not seem related to childcare responsibilities but could be related to more sensitivity to uncertainty during the pandemic.^32^ Amongst those without children, workers who set their own working hours are more likely to have initiated the decision.

**FIGURE 6:**
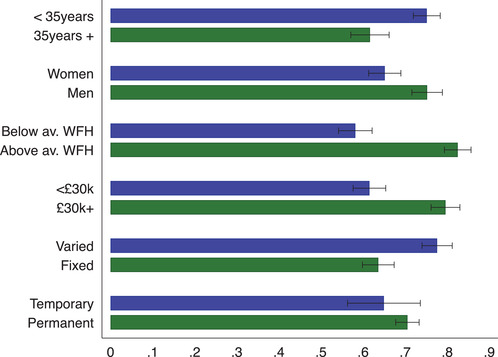
Share of furloughed workers receiving top‐up by individual and job characteristics *Note*: The graph shows the share of workers that are currently furloughed by different individual and job characteristics who report having their salary topped up beyond the 80 per cent subsidy provided by the government. Black bars represent 95 per cent confidence intervals. The sample is restricted to respondents to the April survey wave. ‘Below av. WFH’ are employees who can do less than average tasks from home, while ‘Above av. WFH’ are employees who can do more than average tasks from home. ‘<£30k’ refers to respondents with a yearly gross individual income below £30,000 in 2019, while ‘£30k+’ are those earning more. ‘Varied’ refers to respondents with variable‐hour contracts, while ‘fixed’ refers to those with fixed‐hour contracts.

In principle, the furloughing scheme could result in less pay inequality as it compresses the wage distribution from above by capping the maximum monthly amount at £2,500. However, employers have the choice to top up salaries of furloughed workers above the 80 per cent state contribution or the maximum limit of £2,500, whichever is lowest. In our April survey wave, we asked furloughed respondents whether their employer topped up their salary beyond the level provided by the government. We find that 70 per cent of furloughed workers receive a discretionary salary top‐up by their employer. However, workers on higher incomes are more likely to be topped‐up, reducing the inequality‐reducing effect of the scheme. Figure 6 also shows that (unconditionally) men are more likely to receive discretionary payments.

In the first two columns of Table 3, we analyse heterogeneity in salary top‐ups. In column (1),we see that the probability of receiving a top‐up is decreasing in age and 10 pp lower for women. In column (2), we examine heterogeneity in the probability of receiving a top‐up across the income distribution and by job characteristics. Workers with higher (individual) incomes in 2019 are more likely to receive a top‐up when furloughed. Therefore, the equalising effect of the furloughing scheme is partially mitigated by employers’ decisions to top up their employees' salaries. While the coefficient on gender is insignificant with the full set of controls, we note that it remains positive and significant if only income is controlled for; it is the inclusion of the full suite of job characteristics that reduces the magnitude of the effects. Workers with self‐determined hours are 5 pp more likely to have received a top‐up, perhaps reflecting a reward for greater autonomy (discussed in more detail below).

**TABLE 3:** Terms on which furloughed

	Salary top‐up		Positive work hours		*% usual hours*	
	*(1)*	*(2)*	*(3)*	*(4)*	*(5)*	*(6)*
Age:						
30–39	−0.0227	−0.0042	−0.0802***	−0.0648**	−0.0894***	−0.0676**
	(0.0320)	(0.0308)	(0.0277)	(0.0252)	(0.0332)	(0.0307)
40–49	−0.1353***	−0.0396	−0.2355***	−0.1578***	−0.2789***	−0.1854***
	(0.0405)	(0.0399)	(0.0331)	(0.0313)	(0.0352)	(0.0336)
50–59	−0.1980***	−0.0009	−0.3418***	−0.1841***	−0.4054***	−0.2248***
	(0.0617)	(0.0612)	(0.0441)	(0.0440)	(0.0415)	(0.0427)
60+	−0.3038***	−0.1878*	−0.3981***	−0.2469***	−0.3775***	−0.2114***
	(0.1086)	(0.1078)	(0.0533)	(0.0593)	(0.0538)	(0.0591)
University degree	0.0158	−0.0765***	0.0696***	−0.0160	−0.0012	−0.0642**
	(0.0280)	(0.0287)	(0.0237)	(0.0233)	(0.0262)	(0.0254)
Female	−0.0968***	−0.0104	−0.1949***	−0.0975***	−0.1944***	−0.0952***
	(0.0274)	(0.0289)	(0.0225)	(0.0225)	(0.0253)	(0.0253)
Income 2019 (£10,000s)		0.0138***		0.0154***		0.0148***
		(0.0049)		(0.0040)		(0.0053)
Temporary contract		−0.0243		−0.0177		−0.0307
		(0.0444)		(0.0357)		(0.0396)
Variable hours (worker)		0.0536*		0.1020***		0.1000***
		(0.0325)		(0.0265)		(0.0317)
Variable hours (firm)		−0.0263		0.0472		0.0507
		(0.0379)		(0.0302)		(0.0353)
Non‐salaried contract		0.0599**		0.0590**		0.1206***
		(0.0294)		(0.0244)		(0.0287)
Work from home		0.2878***		0.3272***		0.3690***
		(0.0483)		(0.0402)		(0.0453)
No paid sick leave		−0.3376***		−0.2128***		−0.1928***
		(0.0431)		(0.0306)		(0.0301)
Constant	0.8230***	0.6840***	0.8369***	0.7402***	−0.1710***	−0.6226***
	(0.0547)	(0.1053)	(0.0474)	(0.0860)	(0.0535)	(0.1045)
Observations	1142	1099	1481	1469	1481	1469
$R^{2}$	0.0541	0.2514	0.1835	0.3774	0.1663	0.3589
Region F.E.	Yes	Yes	Yes	Yes	Yes	Yes
Wave F.E.	–	–	Yes	Yes	Yes	Yes
Occupation F.E.	No	Yes	No	Yes	No	Yes
Industry F.E.	No	Yes	No	Yes	No	Yes

*Note*: OLS regressions. Standard errors in parentheses. ^***^ p<0.01; ^**^ p<0.05; ^*^ p<0.1. In columns (1) and (2), the sample is restricted to respondents to the April survey wave who are currently furloughed in their main job. The dependent variable is a dummy variable that takes the value of 1 if the respondent reports that their employer has topped up their salary beyond the 80 per cent funded by the government. Columns (3)–(6) pool responses from the April and May survey waves and restrict the sample to those who are currently furloughed in their main job and report having only one job. The dependent variable in columns (3) and (4) is a dummy variable that takes the value of 1 if the respondent reports positive work hours in the previous week, and 0 otherwise. The dependent variable in columns (5) and (6) is the proportion of weekly hours worked in the previous week compared with typical hours in February.

At the time of our surveys, working was forbidden while currently furloughed. However, 19 per cent of employees in our sample report being explicitly asked to work by their employer despite being currently furloughed. In Figure B.3, we show how this share breaks down by occupation and industry.^33^ There is large variation in the share of furloughed workers who are asked to provide work across occupations. While 44 per cent of furloughed employees working in ‘Computer and Mathematical’ occupations have been asked to work while on furlough, the corresponding share for ‘Transportation and Material Moving’ is 3 per cent. Similarly, 35 per cent of workers in the ‘Information and Communication’ industry report having been asked to work while on furlough, against 8 per cent for ‘Agriculture, Forestry and Fishing’.

Many more furloughed employees report working even if not explicitly compelled to do so by their employer. Two‐thirds of furloughed workers (who only had one job) report having worked a positive amount of hours over the last week. The regression models reported in columns (3)–(6) of Table 3 reveal that women, older workers, and those without paid sick leave are less likely to have continued to work on furlough. Workers on higher incomes but also those on variable‐hour contracts have been more likely to continue working. Those with the flexibility of self‐determined hours (as opposed to those whose schedule is determined by their employer) have been more likely to continue working whilst on furlough, suggesting the importance of worker autonomy in the decision to work whilst furloughed.^34^

**FIGURE 7:**
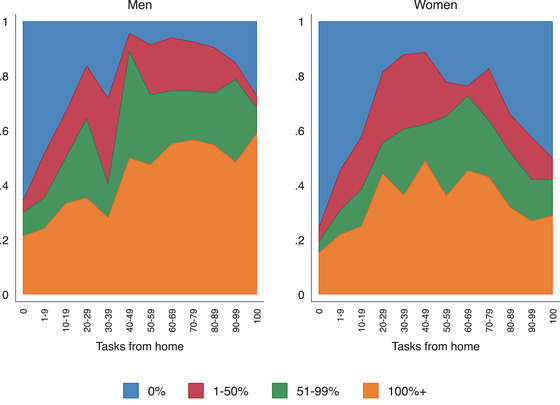
Percentage of usual hours worked while furloughed by the percentage of tasks that can be done from home *Note*: The graph shows the percentage of typical work hours worked in the previous week by respondents who are currently furloughed, by the percentage of tasks that can be done from home. Survey responses for the April and May survey waves are pooled in this figure.

Workers who can do a large percentage of their jobs from home are especially likely to have continued working whilst furloughed (see columns (4) and (6) of Table 3). Figure 7 shows relative hours worked by the percentage of tasks that can be done from home, separately for men (left) and women (right). The relationship is striking. Those who can do the majority of their tasks from home are especially likely to have continued working the same or more hours than usual (orange) while on furlough. The gradient is somewhat less striking for women, perhaps because of caring responsibilities. In Figure B.4, we plot the mean and median hours worked amongst furloughed workers by the percentage of tasks that can be done from home, which confirms the patterns.

On average, including zeros, furloughed workers worked 15 hours (10 hours median). While still substantial, this is a decline in work hours of 44 per cent on average compared with a typical week in February. Although some of these workers might have been furloughed very close to our survey date and therefore might not have been furloughed in the previous week when they report working a positive amount of hours, it is unlikely that this scenario applies to a large fraction of respondents. In Table3, we show how the number of hours worked, despite being furloughed, relates to individual and job characteristics. When controlling for job and individual characteristics, as well as region, industry and occupation fixed effects, we find that women, older workers, those on lower incomes and those without paid sick leave are working fewer hours while currently furloughed.

We note that these patterns cannot be explained by the formal rotation of employees on and off furlough: the CJRS originally allowed workers to work one week in every four‐week period. In our April survey wave, we explicitly asked workers whether their employer was formally rotating them back into work. When we restrict our sample to furloughed employees with a single job who report that their employer is not formally rotating them back into work, we still find that over 60 per cent of furloughed employees report doing some work, with a 42 per cent reduction in weekly hours on average. Table B.1 replicates columns (4) and (6) of Table 3, restricting the sample to furloughed employees who are not being formally rotated into work.

## VI Returning to work and expectations for the future

**FIGURE 8:**
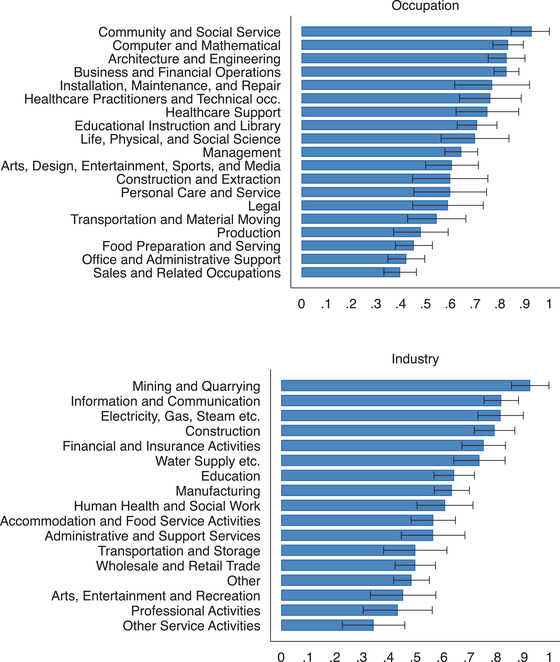
Would accept pay cut to return to work, by occupation and industry *Note*: The graph shows the share of currently furloughed workers who would prefer to go back to work for 80 per cent of their salary instead of staying on furlough, by occupation and industry. Black bars represent 95 per cent confidence intervals. The sample is restricted to furloughed respondents of the May survey wave.

At the time of writing, consumers are being encouraged to leave their homes to spend on the high street and workers are being actively encouraged to return to work.^35^ In our May survey wave, we asked furloughed workers whether they would prefer going back to work for 80 per cent of their salary instead of staying on furlough. On average, 61 per cent of respondents said they would prefer to return to work from furlough even at 80 per cent of pay. However, there are large differences in workers' preferences across occupations and industries (see Figure 8). Workers in service‐sector occupations (e.g. ‘Food Preparation and Serving’ or ‘Sales and Related Occupations'), are significantly less likely to be willing to return to work compared with workers in ‘Computer and Mathematical’ or ‘Architecture and Engineering’ occupations.

In Table 4, we analyse the determinants of workers’ willingness to return to work. Column (1) shows that women are almost 13 pp less likely to report being willing to go back to work for a 20 per cent salary cut, and willingness to return to work decreases with age. In column (2), we analyse heterogeneities in workers' willingness to return to work for a pay cut along the income distribution and for individuals with different contractual arrangements. Workers who can do a larger share of their tasks from home are 17 pp more likely to be willing to go back to work instead of being on furlough. Importantly, individuals employed under variable‐hour work arrangements are significantly more likely to be willing to take a pay cut and return to work, especially for workers who have control of the number of hours they work. This suggests that furloughed workers might value other intangible aspects of their work beyond the monetary compensation.

**TABLE 4:** Prefer to return to work for 80 per cent of pay

	*(1)*	*(2)*	*(3)*
Age:			
30–39	−0.0300	−0.0423	−0.0419
	(0.0412)	(0.0398)	(0.0397)
40–49	−0.1293**	−0.0614	−0.0617
	(0.0506)	(0.0512)	(0.0512)
50–59	−0.1316**	−0.0031	−0.0036
	(0.0609)	(0.0620)	(0.0621)
60+	−0.1845**	−0.0545	−0.0555
	(0.0732)	(0.0798)	(0.0798)
University degree	0.0535	−0.0024	−0.0027
	(0.0347)	(0.0361)	(0.0361)
Female	−0.1302***	−0.0442	−0.0446
	(0.0342)	(0.0349)	(0.0349)
Income 2019 (£10,000s)		0.0163**	0.0163**
		(0.0068)	(0.0068)
Temporary contract		−0.0008	−0.0006
		(0.0600)	(0.0600)
Variable hours (worker)		0.1807***	0.1797***
		(0.0404)	(0.0406)
Variable hours (firm)		0.1142**	0.1130**
		(0.0487)	(0.0490)
Non‐salaried contract		0.0983**	0.0982**
		(0.0391)	(0.0392)
Work from home		0.1709***	0.1710***
		(0.0621)	(0.0621)
No paid sick leave		−0.1327***	−0.1320***
		(0.0438)	(0.0439)
Initiated furlough			0.0097
			(0.0409)
Constant	0.6823***	0.4319*	0.4294*
	(0.0732)	(0.2218)	(0.2220)
Observations	806	801	801
$R^{2}$	0.0744	0.2690	0.2690
Region F.E.	Yes	Yes	Yes
Occupation F.E.	No	Yes	Yes
Industry F.E.	No	Yes	Yes

*Note*: OLS regressions. Standard errors in parentheses. ^***^ p<0.01; ^**^ p<0.05; ^*^ p<0.1. The sample is restricted to currently furloughed respondents in the May survey wave. The dependent variable is a dummy variable that takes the value of 1 if the respondent would prefer to go back to work for 80 per cent of their salary instead of staying on furlough, and 0 otherwise.

Employees who do not have access to paid sick leave beyond the statutory minimum are 13 pp less likely to be willing to return to work for 80 per cent of their salary, even when a rich set of job characteristics are controlled for. This highlights an important trade‐off between health and economic risks; workers without an adequate safety net appear to be more cautious about exposing themselves to health risks at work. Finally, in column (3), we include whether an employee initiated the decision to be furloughed, but we do not find any significant effect.

Despite the government's effort to cushion the negative impact of the coronavirus crisis on the labour market, many workers fear losing their job before August,^36^ and workers who have been put on furlough may feel perilously close to being laid off. In Table 5, we look at workers' expectations about future labour market outcomes. We restrict the sample to individuals who are currently in work and we regress workers' self‐reported probability of losing their job before August on individual and job characteristics, and an indicator for whether they are currently on furlough. Column (1) shows that the expected probability of losing one's job is decreasing in age, and higher for men and workers with a university degree. Column (2) echoes our findings on returning to work and shows that workers with less secure job contracts are more pessimistic about their future labour market outcomes. Notably, workers who can do a large share of their tasks from home find it more likely that they will lose their job before August. In column (3), we examine heterogeneities in the perceived probability of job loss by whether or not workers are currently furloughed. Furloughed workers are much more likely to fear losing their jobs: on average, they report a 15 pp higher likelihood of losing their job before August, controlling for a broad range of individual and job characteristics. Among furloughed workers, those who can do a larger share of their tasks from home are more pessimistic about future employment (see column (4)). For these workers, social‐distancing restrictions on labour supply are unlikely to be the only reason for a low‐productivity match, and thus firm or demand factors could be stronger drivers of subjective expectations of job loss.

**TABLE 5:** Perceived probability of job loss

	In work			Furloughed	Not furloughed
	*(1)*	*(2)*	*(3)*	*(4)*	*(5)*
Age:					
30–39	−0.0316***	−0.0077	0.0073	0.0445***	−0.0229
	(0.0111)	(0.0106)	(0.0103)	(0.0151)	(0.0142)
40–49	−0.1229***	−0.0659***	−0.0448***	−0.0097	−0.0663***
	(0.0117)	(0.0118)	(0.0115)	(0.0195)	(0.0147)
50–59	−0.2033***	−0.1206***	−0.0909***	−0.0409	−0.1077***
	(0.0119)	(0.0126)	(0.0123)	(0.0252)	(0.0150)
60+	−0.2107***	−0.1343***	−0.1128***	−0.0581*	−0.1341***
	(0.0177)	(0.0183)	(0.0175)	(0.0350)	(0.0200)
University degree	0.0172**	0.0089	0.0094	0.0199	0.0091
	(0.0082)	(0.0086)	(0.0083)	(0.0138)	(0.0103)
Female	−0.0581***	−0.0095	−0.0156*	−0.0370***	−0.0032
	(0.0082)	(0.0086)	(0.0082)	(0.0138)	(0.0101)
Income 2019 (£10,000s)		0.0080***	0.0072***	0.0047*	0.0045*
		(0.0019)	(0.0018)	(0.0025)	(0.0025)
Temporary contract		0.0721***	0.0818***	0.0308	0.1154***
		(0.0147)	(0.0147)	(0.0192)	(0.0216)
Variable hours (worker)		0.0493***	0.0359***	0.0165	0.0421***
		(0.0109)	(0.0106)	(0.0160)	(0.0140)
Variable hours (firm)		0.0483***	0.0360***	0.0242	0.0484***
		(0.0130)	(0.0126)	(0.0181)	(0.0178)
Non‐salaried contract		0.0531***	0.0348***	0.0419***	0.0209*
		(0.0098)	(0.0094)	(0.0142)	(0.0124)
Work from home		0.1395***	0.1575***	0.3018***	0.0815***
		(0.0133)	(0.0127)	(0.0254)	(0.0145)
No paid sick leave		−0.0040	−0.0039	−0.0201	0.0042
		(0.0109)	(0.0106)	(0.0186)	(0.0128)
Currently furloughed			0.1554***		
			(0.0087)		
Constant	0.3782***	0.2378***	0.1493***	0.3065***	0.1322**
	(0.0172)	(0.0491)	(0.0451)	(0.0645)	(0.0561)
Observations	4,908	4,877	4,877	1,892	2,985
$R^{2}$	0.0920	0.2178	0.2723	0.2563	0.1814
Region F.E.	Yes	Yes	Yes	Yes	Yes
Wave F.E.	Yes	Yes	Yes	Yes	Yes
Occupation F.E.	No	Yes	Yes	Yes	Yes
Industry F.E.	No	Yes	Yes	Yes	Yes

*Note*: OLS regressions. Standard errors in parentheses. ^***^ p<0.01; ^**^ p<0.05; ^*^ p<0.1. The sample in columns (1)–(3) is restricted to those in work in the April and May survey waves. The sample in column (4) is restricted to those currently on furlough, and in column (5) it is restricted to employees not on furlough. The dependent variable is the respondent's subjective probability of losing their job before 1 August 2020 on a 0–1 scale.

In Table 6, we use data from our May survey wave to examine differences in workers' subjective probability of looking for a new job in the next year. Looking at individual and job characteristics, we find that older workers and workers without a university degree are less likely to look for a new job, whereas workers on temporary contracts report significantly higher likelihoods of job search. Column (3) further shows that furloughed workers are around 10 pp more likely to be currently looking for a job, even when controlling for individual and job characteristics. Interestingly, in all specifications, workers who do not have access to sick pay beyond the statutory minimum report between 4 and 9 pp higher likelihoods of looking for a new job. In column (4), we additionally control for workers' self‐reported probability of job loss before August. As expected, fears of job loss strongly correlate with search behaviour: workers who are more pessimistic about their abilities to retain their job in the short term are significantly more likely to report that they will be looking for a job in the next year. Moreover, once we control for the subjective probability of job loss, we find that the coefficient on the furlough dummy becomes three times smaller, but that it is still significant and around 3 pp. Finally, in column (5), we restrict the sample to workers who reported being furloughed at the time of data collection, and we find that the associations between age, education and on‐the‐job search survive within the sample of furloughed workers.

**TABLE 6:** On the job search

	In work				Furloughed	Not furloughed
	*(1)*	*(2)*	*(3)*	*(4)*	*(5)*	*(6)*
Age:						
30–39	−0.0334*	−0.0104	−0.0058	−0.0079	−0.0340	0.0025
	(0.0180)	(0.0182)	(0.0180)	(0.0172)	(0.0254)	(0.0238)
40–49	−0.0743***	−0.0330	−0.0243	−0.0038	0.0009	0.0023
	(0.0205)	(0.0208)	(0.0206)	(0.0195)	(0.0320)	(0.0256)
50–59	−0.1893***	−0.1276***	−0.1145***	−0.0821***	−0.1309***	−0.0675**
	(0.0217)	(0.0227)	(0.0227)	(0.0210)	(0.0373)	(0.0265)
60+	−0.3143***	−0.2536***	−0.2465***	−0.2069***	−0.2133***	−0.2019***
	(0.0265)	(0.0272)	(0.0268)	(0.0240)	(0.0430)	(0.0309)
University degree	0.0531***	0.0631***	0.0623***	0.0607***	0.0418*	0.0683***
	(0.0140)	(0.0147)	(0.0145)	(0.0137)	(0.0220)	(0.0180)
Female	−0.0146	−0.0099	−0.0139	−0.0002	0.0008	−0.0038
	(0.0136)	(0.0143)	(0.0142)	(0.0134)	(0.0221)	(0.0174)
Income 2019 (£10,000s)		−0.0033	−0.0034	−0.0064**	−0.0058	−0.0078**
		(0.0030)	(0.0030)	(0.0025)	(0.0036)	(0.0035)
Temporary contract		0.0735***	0.0774***	0.0479**	0.0123	0.0682**
		(0.0240)	(0.0240)	(0.0222)	(0.0331)	(0.0314)
Variable hours (worker)		0.0400**	0.0317*	0.0118	0.0490*	−0.0139
		(0.0183)	(0.0181)	(0.0163)	(0.0261)	(0.0219)
Variable hours (firm)		0.0281	0.0221	0.0032	0.0228	0.0049
		(0.0217)	(0.0216)	(0.0203)	(0.0319)	(0.0286)
Non‐salaried contract		0.0371**	0.0269	0.0046	−0.0109	0.0128
		(0.0166)	(0.0164)	(0.0152)	(0.0245)	(0.0206)
Work from home		0.1465***	0.1623***	0.0918***	0.0588	0.1071***
		(0.0217)	(0.0214)	(0.0206)	(0.0395)	(0.0250)
No paid sick leave		0.0706***	0.0671***	0.0615***	0.0840***	0.0423*
		(0.0186)	(0.0184)	(0.0172)	(0.0286)	(0.0224)
Currently furloughed			0.0964***	0.0291**		
			(0.0145)	(0.0137)		
Perceived prob. of job loss				0.4604***	0.4643***	0.4664***
				(0.0249)	(0.0429)	(0.0315)
Constant	0.4394***	0.2650***	0.2114***	0.1335**	0.1977**	0.1541*
	(0.0289)	(0.0808)	(0.0795)	(0.0575)	(0.0855)	(0.0814)
Observations	2,292	2,282	2,282	2,278	800	1,478
$R^{2}$	0.1086	0.1879	0.2029	0.3116	0.3438	0.2882
Region F.E.	Yes	Yes	Yes	Yes	Yes	Yes
Occupation F.E.	No	Yes	Yes	Yes	Yes	Yes
Industry F.E.	No	Yes	Yes	Yes	Yes	Yes

*Note*: OLS regressions. Standard errors in parentheses. ^***^ p<0.01; ^**^ p<0.05; ^*^ p<0.1. The sample in columns (1)–(6) is restricted to those in work in the May survey wave. The sample in columns (6) and (7) is restricted to those currently on furlough and not on furlough, respectively, in the May survey wave. The dependent variable is the respondent's subjective probability of looking for a new job in the next year.

## VII Implications for policy design

Given the high likelihood of future waves of COVID‐19 infection, it is crucial quickly to evaluate the design of the CJRS. It is clear that any future UK policy should allow employees to work on a part‐time basis from the introduction of the scheme. The vast majority of workers report that they can do some of their work tasks from home,^37^ and the majority of workers continued to do some work while on furlough even when this was banned by the scheme. While this has likely introduced inequality between firms that fully complied with the scheme and those that did not, having furloughed employees continue to work is likely to have been welfare‐improving by allowing economic activity to continue.

Preventing work on furlough might also have slowed the adoption of new technologies to enable working from home: why invest in changing work practices if your employees are not supposed to work? In Adams‐Prassl et al. (2020b), we show that improvements in the ability to work from home were the largest in occupations that already had the largest share of workers who could do all tasks from home at the beginning of the crisis. It is plausible that the capacity to work from home could have increased in a wider set of occupations had the furloughing scheme placed fewer restrictions on working.

At the time of writing, the UK government is resisting any extension to the CJRS beyond October 2020. Our results suggest that greater flexibility in the ending of the scheme could be required. Crucially, the duration of the furloughing scheme should be sensitive to continued disruption in schooling and childcare. There is a growing body of evidence that women, and mothers in particular, have been especially hard hit economically by the pandemic.^38^ Even mothers who can work from home face more interruptions to their work time from domestic care responsibilities.^39^ In this paper, we show that mothers have been more likely to request to be furloughed but there is no gender gap for childless workers. There is a real risk that mothers could be forced out of the labour market if the furloughing scheme ends without viable childcare options being available.

Flexibility in the removal of the scheme across different occupations is also warranted. Our results suggest that support for jobs that can be done from home should be phased out more quickly. Furloughed workers who can do a large proportion of their jobs from home are relatively pessimistic about their chance of keeping their job in the medium run. For these workers, social‐distancing measures are unlikely to be the only reason for a low‐productivity match and they should not be prevented from moving to more viable firms. However, in jobs that are relatively difficult to do from home, labour supply restrictions from social‐distancing measures should be taken into consideration, as the match might be efficient outside a pandemic.

Returning to work outside the home brings more opportunities for exposure to, and transmission of, the virus. While the majority of furloughed workers would prefer to return to work even at 80 per cent of their usual pay, workers without employer‐provided sick pay have a significantly lower willingness to pay to return to work. Worryingly, we find that workers without additional sick pay are significantly more likely to continue to work even with mild coronavirus symptoms (Table B.2 in the online Appendix). The UK has one of the least generous statutory sick pay schemes in Europe, which was described as ‘manifestly inadequate’ by the European Committee of Social Rights (2017). Complementing findings from causal studies of changes in sick pay coverage,^40^ our results suggest that the provision of more generous sick pay could help to support the economic recovery by encouraging workers to return to work while infection rates remain above zero, and supporting sick workers to take time off work when they pose a risk to others.

## VIII Conclusion

In this paper, we exploit survey data from the UK to document differences in furloughing under the Coronavirus Job Retention Scheme across job and individual characteristics. We show that, while a significant proportion of workers in our sample are currently on furlough, there are large differences in the use of the furloughing scheme across industries and occupations. Further, we document that women, younger workers, and workers with alternative work arrangements have been more likely to be put on furlough.

Relatedly, we provide evidence of differences in the terms under which employees have been furloughed. In particular, our analysis shows that a significant proportion of workers who are on furlough still report working a positive amount of hours. Further, the number of hours worked while on furlough is increasing in the share of tasks that workers can perform from home, and is higher for respondents whose employer agreed to top up their wage beyond the state contribution of 80 per cent. Finally, we show that being on furlough is associated with higher self‐reported probabilities of job loss before August for respondents who are in paid work at the time of data collection, and a higher likelihood of searching for a new job.

Our results highlight the benefits of allowing employees to work while enrolled in a STW scheme and the need for flexibility in the duration of government support across occupations and in response to childcare disruption. Finally, our results suggest that the provision of more generous sick pay could help to support the economic recovery by encouraging workers to return to work while infection rates remain above zero, and supporting sick workers to take time off work when they pose a risk to others.

For future research it will be important, but challenging, to understand what would have happened to the UK economy under alternative policy responses or with no furloughing scheme at all. This understanding could contribute to the design of STW schemes that are kept in place to help stabilise the economy in response to large negative exogenous shocks with mechanisms that contain uncertainty and increase efficiency.

## Supporting information

Online Appendix

## Footnotes

1 Giupponi and Landais, 2020.

2 Source: HMRC coronavirus (COVID‐19) statistics.

3 These figures exclude furloughed employees who said they were being formally rotated back into work by their employer.

4 Adams‐Prassl et al., 2020b.

5 Giupponi and Landais, 2020.

6 See, for example, Giupponi and Landais (2020), Cahuc, Kramarz and Nevoux (2018) and Kopp and Siegenthaler (2018).

7 See, for example, Blundell et al. (2020), Benzeval et al. (2020) and Piyapromdee and Spittal (2020).

8 Alon et al., 2020; Dingel and Neiman, 2020; Mongey and Weinberg, 2020.

9 Pichler and Ziebarth, 2020; Marie and Vall Castelló, 2020; Adams‐Prassl et al., 2020a.

10 Short‐time work dates back to 1910 when it was first used in the mining industry.

11 The usual replacement rate is 67 per cent for employees with children. During the COVID‐19 pandemic, the replacement rate has been increased to 70 per cent (or 77 per cent for employees with children) for those working half‐time from the fourth month onwards, and to 80 per cent (or 87 per cent for employees with children) from the seventh month onwards.

12 See Schulten and Müller (2020) for differences in the regulations across European countries. Some US states also have short‐time compensation (STC) schemes. STC programmes are implemented at the state level and there are differences among state programmes.

13 Giupponi and Landais, 2020.

14 See, for instance, Rosenberg (2020).

15 Schulten and Müller, 2020.

16 From 30 June onwards, employers were only able to furlough employees that they had furloughed for a full three‐week period at any time between 1 March and 30 June 2020. Thus, the final date by which an employer could have furloughed an employee for the first time was 10 June 2020.

17 An employee could be furloughed and do no work for three weeks, and then be brought off furlough to work for the employer for a one‐week period before potentially being put back on furlough. However, furloughed employees can take on a new job with a different employer, provided this is permitted by their contract of employment in general.

18 In August, the government contribution towards the employee's pay when on furlough remains at 80 per cent but employers are required to pay employer national insurance and pension contributions. In September, employers are also required to pay 10 per cent of wages and the government contributes 70 per cent. In October, the employer contribution increases to 20 per cent with the government contribution falling to 60 per cent.

19 Online Appendix C includes the questionnaire.

20 The survey was scripted in the online survey software Qualtrics. Participants received modest incentives for completing the survey.

21 We deliberately chose to survey new participants in the second survey wave (i.e. there are no participants who participated in the survey more than once).

22 Respondents who had no job in the week before the survey were asked analogous questions about their last job.

23 Gardiner and Slaughter, 2020.

24 Both our surveys took place before the announcement of flexible furlough.

25 The UK Office for National Statistics estimates there were 27.7million employees in their February 2020 labour market bulletin. See https://www.ons.gov.uk/employmentandlabourmarket/peopleinwork/employmentandemployeetypes/bulletins/employmentintheuk/february2020.

26 Most occupations and industries saw little change in the share of furloughed workers across these survey waves (see Figure B.1 in the online Appendix). Meaningful easing of lockdown did not begin until 4 July in many sectors.

27 Figure B.2 in the online Appendix shows the share of employees that have lost their job, have been furloughed, and have remained employed and not furloughed by occupation and industry.

28 We note that some differences between regions remain significant, even when controlling for job and individual characteristics.

29 Both our survey waves took place before the introduction of flexible furloughing.

30 See Section III.

31 Adams‐Prassl et al., 2020c; Andrew et al., 2020; Biroli et al., 2020; Farré et al., 2020; Sevilla and Smith, 2020.

32 Interactions between gender and hours arrangements are insignificant.

33 We exclude employees who report they are being formally rotated into work.

34 Mas and Pallais, 2020.

35 See, for example, the introduction of the ‘Eat Out to Help Out’ scheme on 8 July 2020: https://www.gov.uk/guidance/get-a-discount-with-the-eat-out-to-help-out-scheme.

36 Adams‐Prassl et al., 2020c.

37 Adams‐Prassl et al., 2020b.

38 Adams‐Prassl et al., 2020c; Andrew et al., 2020; Benzeval et al., 2020.

39 See, for example, Adams (2020) and Andrew et al. (2020).

40 Marie and Vall Castelló, 2020; Pichler and Ziebarth, 2020.
