# Supplementary material for: Furloughing
Source: Fisc Stud. 2020 Nov 30;41(3):591–622. doi: 10.1111/1475-5890.12242 (PMC7753414; doi:10.1111/1475-5890.12242)
Supplement: Supplementary file 1 — Online Appendix [file FISC-41-591-s001.pdf]

Furloughing: Online Appendix

Abi Adams-Prassl, Teodora Boneva, Marta Golin and Christopher Rauh

A. Data Description

Table A.1: *Distribution across regions*

Region	National	April	May
Scotland	8.42	8.54	8.48
Northern Ireland	2.76	2.80	2.74
Wales	4.79	4.87	4.79
North East	4.06	4.12	4.04
North West	11.00	11.11	10.95
Yorkshire and the Humber	8.24	8.34	8.21
West Midlands	8.80	8.92	8.78
East Midlands	7.27	7.38	7.26
South West	8.59	8.70	8.61
South East	13.70	13.87	13.69
East of England	9.29	8.03	9.30
Greater London	13.15	13.32	13.15
Observations		4,931	4,009

Note: National figures refer to the latest available estimates for the population of residents aged 18 or above and come from the Office for National Statistics.
Source: Office for National Statistics (2019), "Estimates of the population for the UK, England and Wales, Scotland and Northern Ireland". Data retrieved from `https://www.ons.gov.uk/peoplepopulationandcommunity/populationandmigration/populationestimates/datasets/populationestimatesforukenglandandwalesscotlandandnorthernireland`.

Table A.2: *Background characteristics*

	LFS	April	May
Female	0.47	0.552	0.550
University	0.357	0.488	0.464
Aged <30	0.232	0.281	0.283
Aged 30–39	0.230	0.333	0.264
Aged 40–49	0.217	0.238	0.196
Aged 50–59	0.217	0.114	0.163
Aged 60+	0.104	0.033	0.095

Notes: The table shows the mean demographic characteristics of economically active individuals in the UK. These were calculated using the frequency weights provides in the Labour Force Survey (LFS). The unweighted averages of these demographic variables in our survey waves are also reported.

B. Additional results

Figure B.1: *Share of furloughed workers by occupation and industry across survey waves*

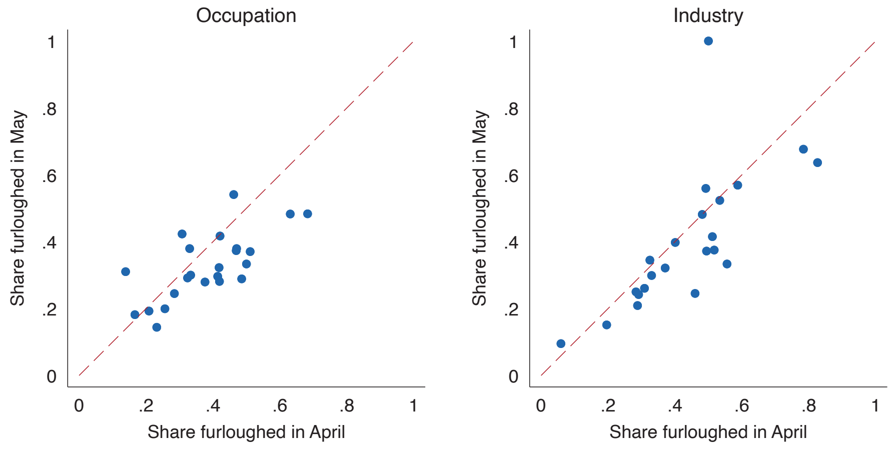

Note: The graph shows the share of workers that are furloughed by occupation and industry, separately for the April (x-axis) and May (y-axis) survey wave. Each dot represents one occupation (left) or industry (right).

Figure B.2: *Employment status by occupation and industry*

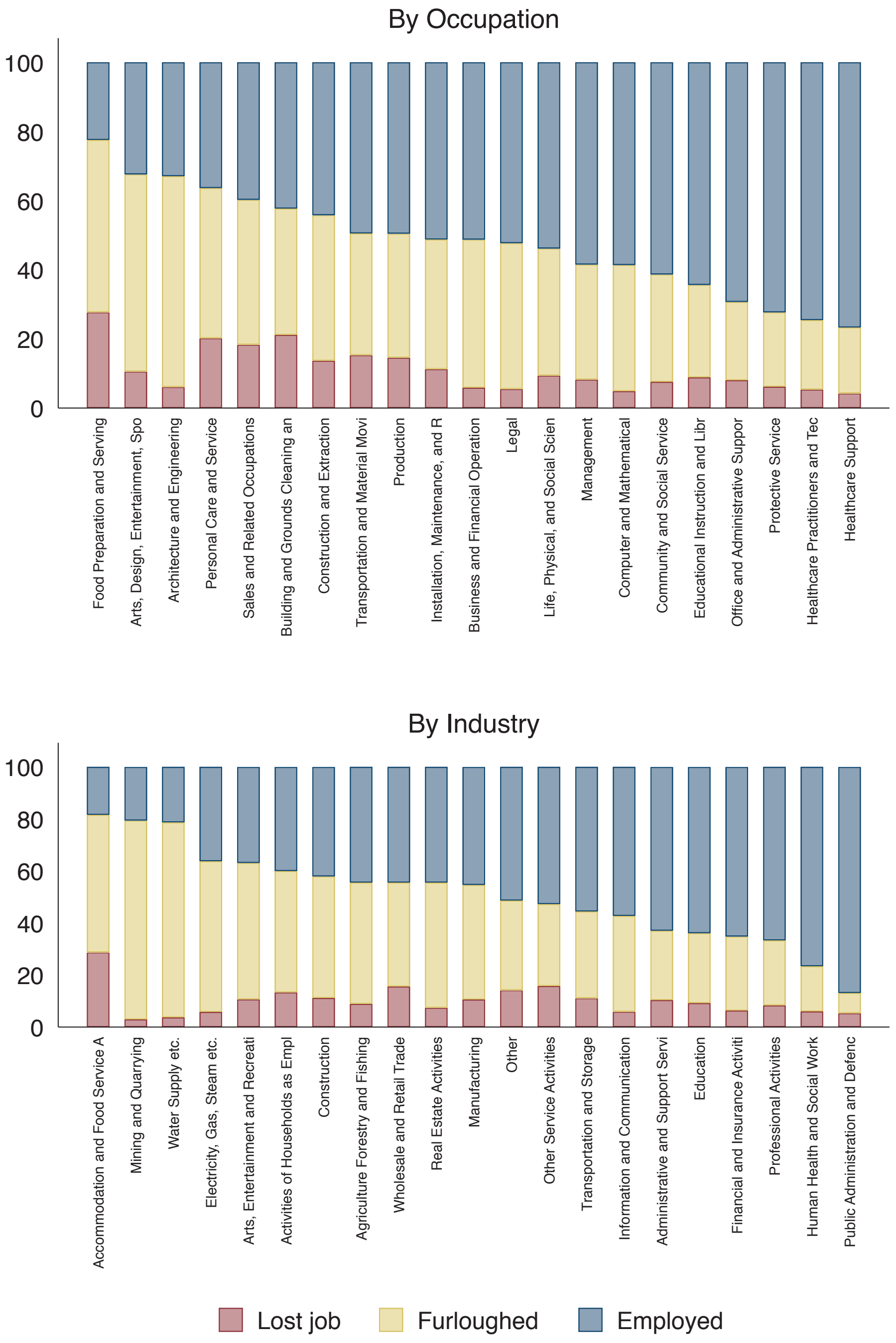

Note: The figure shows the share of workers who are employed (blue), furloughed (yellow) or have lost their job due to the COVID-19 crisis (red), by occupation (top) and industry (bottom). Survey responses for the April and May survey waves are pooled in this figure.

Figure B.3: *Share of furloughed workers being asked to work*

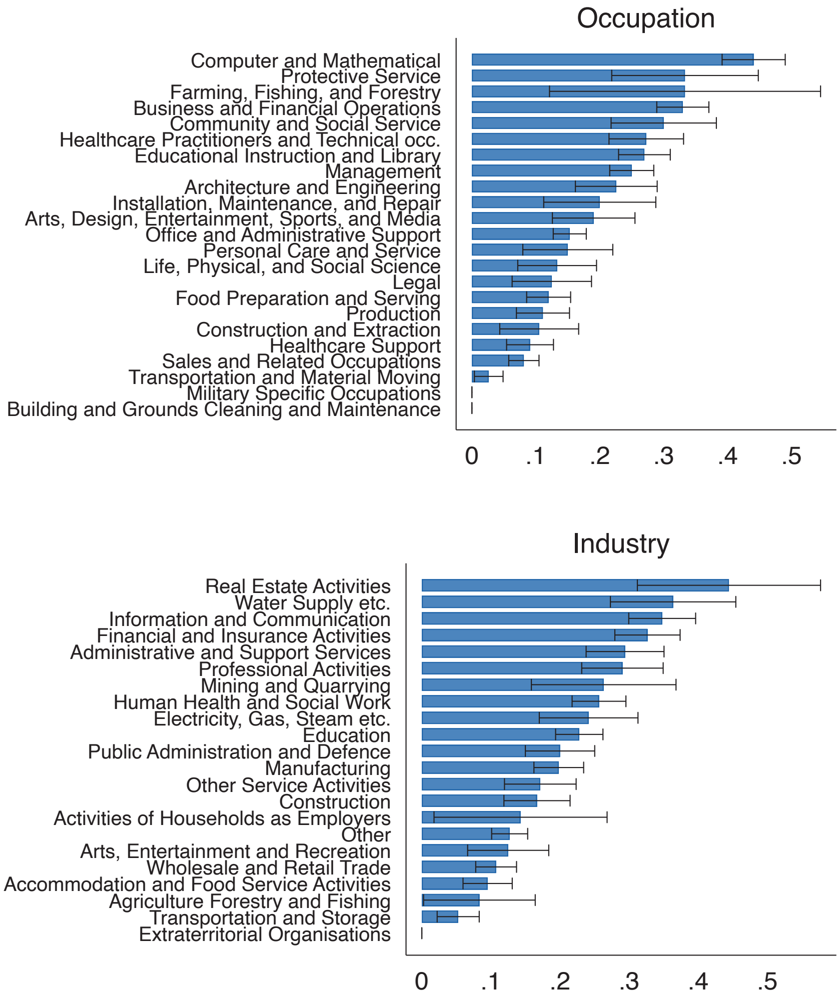

Note: The sample is restricted to respondents to the April survey wave. The horizontal bars show the average share of furloughed workers who report having been asked to work while on furlough for each occupation (top) and industry (bottom). The black bars represent 95 per cent confidence intervals.

Figure B.4: *Percentage of usual hours worked while furloughed by percentage of tasks that could be done from home*

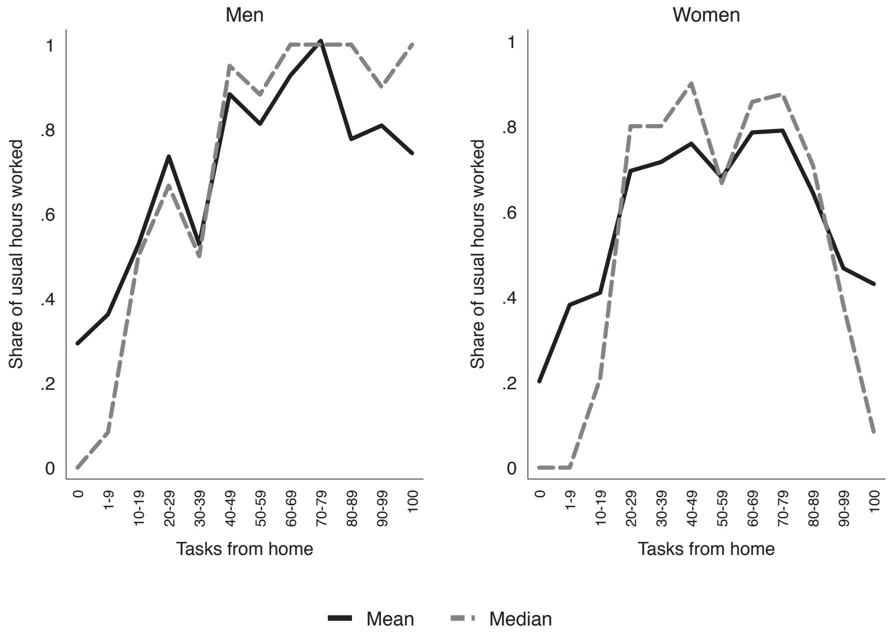

Note: The graph shows the mean and median percentage of typical work hours worked last week by respondents who are currently furloughed by the quintiles of the percentage of tasks one can do from home. Survey responses for the April and May survey waves are pooled in this figure.

Table B.1: *Hours worked while on furlough: sensitivity to formal workplace rotation*

	Positive work hours		% usual hours	
	(1)	(2)	(3)	(4)
Age:				
30–39	-0.1100^{***}	-0.1398^{***}	-0.1524^{***}	-0.1787^{***}
	(0.0335)	(0.0420)	(0.0421)	(0.0462)
40–49	-0.1532^{***}	-0.1766^{***}	-0.2252^{***}	-0.2346^{***}
	(0.0405)	(0.0513)	(0.0451)	(0.0514)
50–59	-0.1299^{**}	-0.1685^{**}	-0.2491^{***}	-0.2573^{***}
	(0.0615)	(0.0702)	(0.0614)	(0.0655)
60+	−0.0934	−0.0942	−0.1271	−0.0928
	(0.1312)	(0.1367)	(0.1307)	(0.1332)
University degree	-0.0779^{**}	-0.0834^{**}	-0.1316^{***}	-0.1149^{***}
	(0.0303)	(0.0371)	(0.0347)	(0.0383)
Female	-0.0817^{***}	-0.0884^{**}	-0.0926^{***}	-0.0736^{*}
	(0.0287)	(0.0369)	(0.0348)	(0.0396)
Income 2019 (£10,000s)	0.0223^{***}	0.0263^{***}	0.0184^{***}	0.0241^{***}
	(0.0046)	(0.0066)	(0.0065)	(0.0078)
Temporary contract	0.0471	0.0413	−0.0138	0.0049
	(0.0455)	(0.0543)	(0.0486)	(0.0552)
Variable hours (worker)	0.0627^{*}	0.0488	0.0459	0.0416
	(0.0336)	(0.0443)	(0.0417)	(0.0476)
Variable hours (firm)	0.0464	0.0498	0.0231	0.0576
	(0.0409)	(0.0530)	(0.0512)	(0.0590)
Non-salaried contract	0.0355	0.0509	0.1154^{***}	0.1023^{**}
	(0.0316)	(0.0414)	(0.0376)	(0.0442)
Work from home	0.2991^{***}	0.3075^{***}	0.3405^{***}	0.2808^{***}
	(0.0502)	(0.0614)	(0.0586)	(0.0658)
No paid sick leave	-0.1861^{***}	-0.1626^{***}	-0.2034^{***}	-0.1664^{***}
	(0.0455)	(0.0473)	(0.0448)	(0.0460)
Constant	0.7835^{***}	0.7914^{***}	-0.5339^{***}	-0.4612^{***}
	(0.0975)	(0.1137)	(0.1244)	(0.1253)
Observations	823	653	823	653
R^2	0.3397	0.3354	0.3431	0.3466
Region fixed effects	Yes	Yes	Yes	Yes
Occupation fixed effects	Yes	Yes	Yes	Yes
Industry fixed effects	Yes	Yes	Yes	Yes

Note: OLS regressions. Standard errors in parentheses. $^{***}p < 0.01$; $^{**}p < 0.05$; $^{*}p < 0.1$. All specifications restrict responses to April survey wave. Columns (1) and (3) restrict the sample to those who are currently furloughed in their main job and report having only one job. In columns (2) and (4), the dependent variable is further restricted to those who did not report being formally rotated back into work.

Table B.2: *Working with cold-like symptoms*

	(1)	(2)	(3)
Age:			
30–39	0.0540**		0.0215
	(0.0241)		(0.0250)
40–49	0.1388***		0.0917***
	(0.0253)		(0.0272)
50–59	0.1563***		0.0995***
	(0.0308)		(0.0334)
60+	0.0354		−0.0110
	(0.0586)		(0.0592)
University degree	0.0274		−0.0031
	(0.0184)		(0.0206)
Female	0.0299		0.0101
	(0.0183)		(0.0205)
Income 2019 (£10,000s)		−0.0094**	−0.0098**
		(0.0041)	(0.0043)
Temporary contract		−0.1284***	−0.1172***
		(0.0344)	(0.0344)
Variable hours (worker)		−0.0120	−0.0065
		(0.0251)	(0.0251)
Variable hours (firm)		−0.0872***	−0.0796**
		(0.0315)	(0.0315)
Non-salaried contract		−0.0690***	−0.0603***
		(0.0229)	(0.0232)
Work from home		−0.0042	0.0061
		(0.0312)	(0.0314)
No paid sick leave		0.0716***	0.0592**
		(0.0250)	(0.0252)
Constant	0.6267***	0.9045***	0.8534***
	(0.0372)	(0.1063)	(0.1103)
Observations	2,660	2,611	2,611
R^2	0.0308	0.0795	0.0861
Region fixed effects	Yes	Yes	Yes
Occupation fixed effects	Yes	Yes	Yes
Industry fixed effects	Yes	Yes	Yes

Note: OLS regressions. Standard errors in parentheses. $^{***}p < 0.01$; $^{**}p < 0.05$; $^{*}p < 0.1$. Sample restricted to employees in the April wave and the dependent variable is a dummy variable that takes the value of 1 if the respondent reports that they would definitely or probably work with cold-like symptoms.

C. Questionnaire

Employment status and hours worked

How many jobs, where self-employment activity counts as a job, did you have in February 2020? Please think of any work you did other than completing surveys. If you were furloughed from a job, please count this as a job.
Many people work as employees, where they have an employment contract with an employer, or in self-employment. There is a lot of variation in self-employment, some people might be selling goods or services in their own business, or working through a digital platform such as Uber or Upwork. In addition to working a regular job for an employer, sometimes people do other things to earn money. These activities also count as self-employment.
[None, 1, 2, 3 or more]

[If worked at least one job in February] *Think about a typical week in February for you at work (in all of your jobs). How many hours did you work in a typical week in February?* [Answers in five-hour increments, from 0 to "More than 55 hours"]

How many jobs, where self-employment activity counts as a job, have you had last week? Please think of any work you did other than completing surveys. If you were furloughed from a job, please count this as a job.
Many people work as employees, where they have an employment contract with an employer, or in self-employment. There is a lot of variation in self-employment, some people might be selling goods or services in their own business, or working through a digital platform such as Uber or Upwork. In addition to working a regular job for an employer, sometimes people do other things to earn money. These activities also count as self-employment.
[None, 1, 2, 3 or more]

[If worked at least one job last week] *Now think about all the work you did last week (in all of your jobs). How many hours did you work last week?* [Answers in five-hour increments, from 0 to "More than 55 hours"]

[If reports working zero jobs last week] *Please think about your last job. In your last job, were you working as an employee or self-employed?* [Employee, Self-employed]

[If reports working at least one job last week] *In your main job, that is the job that you usually spend the most time working in, are you working as an employee or self-employed?* [Employee, Self-employed]

[For current employees] *Have you been furloughed?*[1] [Yes, No]

[For furloughed employees – April wave] *For the period you are being furloughed, has your employer agreed to top up the government wage support? The government will pay 80% of furloughed employees' wages up to a maximum of £2500 per month. Some employers might choose to top up the scheme so that employees receive more than 80% of their usual wages.* [Yes, No]

[For furloughed employees – April wave] *During the period you have been furloughed, have you still been asked to do any work for your employer?* [Yes – I have been asked to do work while still furloughed, Yes – I have been formally rotated back into work, No]

[For furloughed employees – May wave] *Was the decision to be furloughed...?* [Five-point scale from "Fully your employer's decision" to "Fully your decision"]

[For furloughed employees - May wave] *If you could go back to work the same number of hours as you did on a typical week in February but be paid 80% of your salary, would you prefer going back to work rather than staying on furlough?* [Yes – I would prefer going back to work, No – I would prefer staying on furlough]

[If reports working zero jobs last week] *For how long have you not had a job?* [Recorded in weeks/months]

[If reports working zero jobs last week] *If you lost your job recently, do you think this was related to the coronavirus outbreak?* [Answers on five-item scale, from "Definitely yes" to "Definitely no", with additional option "I did not lose my job recently"]

How likely is it that you will look for a new job in the next 12 months?[Answer on a continuous 0–100 scale]

Income

Which category represents your total individual annual income (before taxes)

[1] In the May survey wave, the answer options were [Yes – I am currently on furlough, Yes – but I am no longer on furlough, No].

in 2019? This should include money from all jobs, net income from a business or farm, and any rent, pensions, dividends, interest, social security payments or other money income you received. [Answers on 12-point scale, from "Less than $10,000" to "$150,000 or more"]

Job characteristics: Questions phrased to refer to main or last job, depending on the respondent's employment status.

What sort of occupation best describes this job? [O*NET SOC 2018 major groups]

What category best describes the industry you work in? [NACE Rev. 2 industry classification]

[For current or former employees] *Do you have a permanent contract?* [Yes, No]

[For current or former employees] *Is your job salaried or how do you get paid?* [Salaried, Hourly, Paid by the job, Commission or tips only, Other]

[For current or former employees] *Are the number of hours you work fixed or do they vary?* [Fixed, Vary – I choose how many hours I work, Vary – My employer decides how many hours I work but I am guaranteed some work each week, Vary – I am an on-call worker]

In your job, what percentage of the tasks could you do from home? Examples: Andy is a waiter and cannot do any of his work from home (0%). Beth is a website designer and can do all her work from home (100%). [Answer on 0-100 slider]

[For current or former employees] *In addition to statutory sick pay, how many days of paid sick leave are you entitled to per year through this job?* [None, 1–5 days, 6–10 days, 11–15 days, 16–20 days, More than 21 days]

Expectations
On a scale of 0–100%, how likely are the following scenarios to occur before 1 August 2020?

- *I will lose my job or shut my business if self-employed*
